# Thermomechanical Stability of Carbyne-Based Nanodevices

**DOI:** 10.1186/s11671-017-2099-4

**Published:** 2017-05-04

**Authors:** Sergiy Kotrechko, Andrei Timoshevskii, Eugene Kolyvoshko, Yuriy Matviychuk, Nataliya Stetsenko

**Affiliations:** 1 G.V. Kurdyumov Institute for Metal physics, Kyiv, Ukraine; 2 Taras Shevchenko Kyiv National University, Kyiv, Ukraine

**Keywords:** Carbyne, Carbyne-based nanodevice, Service time, Thermally induced instability, Polyyne-cumulene transition

## Abstract

An approach is developed to predict stability of carbyne-based nanodevices. Within this approach, the thermo-fluctuation model of instability and break of contact bond in nanodevices, containing carbyne chains and graphene sheets, is offered. Unlike the conventional models, it does not include empirical constants. The results of DFT calculations are used as initial data for this model. Possibility of synergistic effect of temperature and mechanical load on stability and value of service time of carbyne-based nanodevices is predicted. It is ascertained, that this synergism results in a significant (by many orders of magnitude) decrease in the lifetime of nanodevices containing carbyne chains. The atomic mechanism of this phenomenon is outlined. Conditions of thermo-force loading are predicted at which a service time of these devices is sufficient for applications.

## Background

Creation of all-carbon-based nanodevices is currently one of the promising directions in development of nanoelectronics [1–3]. The use of monatomic chains of carbon atoms (carbynes) is a characteristic feature of these devices. This will enable not only to employ the unusual functional properties of carbynes, but also to approach the maximum attainable level of miniaturization of these devices. At that, stability and service time of such devices is one of the key challenges towards creating all-carbon-based nanodevices. The results of direct experimental tests of carbyne for tension [4], as well as ab initio simulation findings [5] showed that carbyne has an extremely high level of strength, which is more than 2 times higher than strength of graphene that is still considered as the strongest material in the world [6]. However, break of only one atomic bond is enough for the failure of such nanodevices. This requires the development of innovative methods of diagnostics and prediction of the lifetime of such devices, which differ fundamentally from currently existing approaches. This is due to the fact that in the present case the characteristics of interatomic interaction must influence directly the lifetime of the nanodevices. On the one hand, this creates considerable difficulties for the experimental methods of determining their reliability, but on the other hand, it offers significant opportunities for using calculation methods.

Recently, molecular dynamic (MD) simulation seems a direct approach to the problem, but unfortunately it covered timescale, which does not exceed several microseconds. Therefore, it becomes necessary to develop analytical methods for predicting the reliability of such devices over long time intervals of their operation, which are estimated to be the years. The research results [7] can be considered as one of the attempts aimed at the development of such methods. In this work, carbyne chain connecting two graphene sheets was considered as a model element of such nanodevice. To predict the lifetime of carbyne nanoconductor, energy approach was employed in this work. According to this approach, break of the atomic bond is associated with thermally activated overcoming of the barrier whose height equals the binding energy. It was shown that under certain conditions the proposed model produces the Arrhenius form of the transition theory [8, 9] and the Meyer–Neldel compensation rule [10, 11]. However, in general, the model gives more accurate results than the transition state theory.

Generally, the energy approach is convenient for the description of thermally activated processes of defects formation or fracture of *unloaded* solids. However, accounting for the action of mechanical stresses in this approach is associated with considerable difficulties, since it requires knowledge of regularities governing the effect of these stresses on the energy barrier height [12].

Significant temperature gradients as a result of the different electrical conductivity of graphene sheet and carbyne may be the reason for mechanical stresses in carbyne-based nanodevices. Moreover, deformation can be created artificially to change functional properties of carbyne since carbyne tension results in a change of the band gap [13]. It enables to use the chains of carbyne as the elements of nanoscale lasers and other optoelectronic devices with tunable wavelengths [14]. Thus, it becomes necessary to develop a general approach to describe the stability of carbyne-based nanodevices under simultaneous influence of temperature and mechanical stresses.

The present article gives a brief description of the fluctuation model of atomic bond instability in a one-dimensional crystal and demonstrates the possibility of using it to predict the thermo-mechanical stability of carbyne-based nanodevices. Based on the results of DFT computation, the atomistic interpretation of the synergistic effect of temperature and mechanical load is given.

## Methods

**Fig. 1:**
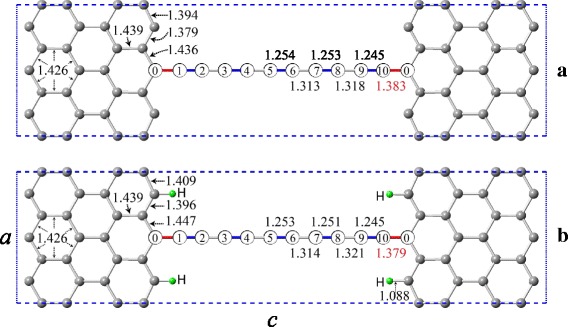
Elementary cell - *a* = 7.41 Å, *c* = 29.98 Å. Thickness of the cell is 10 Å. (bond lengths in the figure are in Å): **a** – without hydrogen; **b** – four bonds terminated with hydrogen

Nanodevice is composed of two infinite half-planes of graphene connected by the carbyne chain containing 10 atoms. Figure 1a presents the elementary cell of the ordered superstructure which was used to simulate the nanodevice. This superstructure consists of two infinite half-planes of graphene connected by the periodically located carbyne chains. A distance between the carbyne chains is equal to the cell parameter *a*. The value of this parameter was selected to eliminate interaction between the chains.

The configuration of carbyne-based nanodevice, considered in this work, is commonly used in solving problems of their lifetime prediction [7]. In such a nano-element, there are “dangling” bonds of carbon atoms on the boundaries of the graphene sheet. In real nanodevices, these bonds can be terminated by hydrogen atoms. Therefore, in this paper a model “graphene-carbyne-hydrogen” system was additionally investigated (Fig. 1b).

The mechanical properties and total energies of this object were calculated using the *Quantum*-ESPRESSO (QE) program package [15], which uses planewaves as a basis set. The GGA-PBE [16] exchange-correlation potential was employed. The ultrasoft pseudopotential for carbon, generated according to *Vanderbilt* scheme (C.pbe-van_ak.UPF, code version 7.3.4) (http://www.quantum-espresso.org/) was used. The calculations applying this potential were earlier used to simulate the structure and mechanical properties of carbyne chains of different length containing even and odd number of atoms [5]. The results of these studies demonstrated that the use of pseudopotential ensures both the required accuracy of calculation of the total energy of carbyne and obtaining the reliable information on carbyne atomic structure and mechanical properties, in particular, reproduction of structure of cumulene and polyyne. Moreover, the use of this pseudo-potential in the present study will enable to compare correctly the properties of isolated carbyne chains with the properties of carbyne chains connecting the graphene sheets.

The pseudo-potential of hydrogen was generated according to the Troullier-Martins scheme using the program package Fhi98PP (H.pbe-mt_fhi.UPF) (http://www.quantum-espresso.org/).

It was used a 60 Ry kinetic energy cutoff for wavefunction, and a 4 × 1 × 4 Monkhorst-Pack [17] mesh for the sampling of the Brillouin zone. Two graphene half-planes connected by carbon chain consisting of 10 atoms, were simulated by a superstructure containing 58 atoms per unit cell (Fig. 1). The cell parameter *a =* 7.41 Å was inherited during cell design from pure graphene sheet calculated within this work. The vacuum separation between the sheets (cell parameter *b*) equals to at least 10 Å, which allows to eliminate interaction between neighbour sheets. The total energy convergence criterion of the self-consistent field procedure was set to 1.0 × 10^−6^ Ry. Lattice constant *c* and atomic positions in cell were optimized using the Broyden-Fletcher-Goldfarb-Shanno (BFGS) algorithm [18, 19]. Structural optimizations were performed until the forces acting on atomic nuclei became less than 0.001 Ry/a.u. Thus, the obtained unit cell parameter *c* equals to 29.98 Å in an unloaded state.

Uniaxial tension of model structure in direction of the *c*-axis was simulated.

The value of force *F* acting on the chain was calculated as:

1 $$ F=\frac{dE}{dc} $$

where *E* is the total energy; *c* is the unit cell parameter for model structure along *c*-axis (Fig. 1).

Based on these data, we have built a dependence of the force acting in the contact bond on its length.

### Fluctuation Model

Lifetime of nanodevices containing monatomic chains is predetermined by the waiting time of break of a contact bond (Fig. 1). As noted above, a classical approach to this problem consists in using the Arrhenius equation, or its later modifications. In this case, the probability of atomic bond break is equivalent to probability of appearance of fluctuation of the atom kinetic energy, sufficient to overcome the energy barrier which magnitude is equal to the binding energy. This takes place in the case of an unloaded crystal. For the general case of a mechanically loaded crystal the probability of an atomic bond break can be formulated as follows:

2 $$ P\left(\delta \ge {\delta}_c\right) = {P}_c $$

where *P*_*c*_ is the probability of failure; *δ*_*c*_ is the critical value of bond length fluctuation *δ*.

**Fig. 2:**
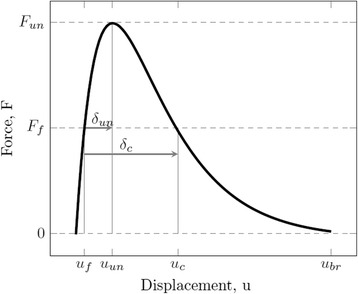
Strain diagram for contact bond (scheme): *F*_*f*_ is the value of “applied” force; *u*_*f*_ is the deviation of an atom from the equilibrium position due to mechanical load; *F*_*un*_ and *u*_*un*_ are the force and displacement of a contact bond instability, respectively; *δ*_*un*_ is the fluctuation of the bond instability; *u*_*c*_ and *δ*_*c*_ are the critical displacement and critical fluctuation, respectively; *u*_*br*_ is the displacement of bond break.

In our case *δ*_*c*_ characterizes critical increasing in the length of a contact bond, which, eventually, gives rise to break of this bond. Dependence of *δ*_*c*_ on applied load *F*_*f*_ is schematically shown in Fig. 2 by the example of pair approximation for the atomic interaction. According to this figure, the value of critical fluctuation *δ*_*c*_ is predetermined by the level of applied load *F*_*f*_.

Reaching of the limit value *F*_*un*_ by *F*_*f*_ is the condition for non-fluctuation (athermal) failure. In this case, magnitude of the force *F*_*f*_ is sufficient not only to induce instability of the atomic interaction, but also to break the atomic bond after that. To initiate the bond break at lower loads, thermal fluctuations are required. However, to ensure that these fluctuations have caused not only a *short-term* instability of the atomic interaction, but also a subsequent bond break, the atomic displacement should not be less than the *critical* value *u*_*c*_, which depends on both the applied load *F*_*f*_ and the intensity of decrease in the force of atomic interaction for displacements *u*_*un*_ ≤ *u* ≤ *u*_*br*_ (Fig. 2). With a decrease in the applied load *F*_*f*_ the required value of a critical fluctuation *δ*_*c*_ increases.

In general, the fluctuation value may be defined as:

3 $$ \delta = u-{u}_f $$

where *u*_*f*_ is the value of displacement due to applied load *F*_*f*_ (Fig. 2).

In *general case*, the dependence of energy of atomic interaction, *E*(*u*), on the atomic displacement value can be represented with sufficient accuracy as:

4 $$ E(u)-{E}_0\approx \frac{\beta {u}^2}{2}-\frac{\gamma {u}^3}{3}+\frac{\eta {u}^4}{4} $$

where *E*_0_ is the binding energy; *β*, *γ*, and *η* are the coefficients.

Accordingly, fluctuations in energy will be the following:

5 $$ \varepsilon \left(\delta \right)= E\left({u}_f+\delta \right)- E\left({u}_f\right), $$

and the density distribution of fluctuations, *g*(*δ*), can be described with sufficient accuracy by the Boltzmann distribution:

6 $$ g\left(\delta \right)=\frac{1}{Z} \exp \left[-\frac{\varepsilon \left(\delta \right)}{k_B T}\right] $$

where *Z* is the statistical weight; *k*_*B*_ is the Boltzmann constant; *T* is the temperature.

Accounting for (5), the expression for the probability of a bond breaking, i.e., of realization of critical fluctuation, *δ*_*c*_, takes the form:

7 $$ P\left(\delta >{\delta}_c\right)=\frac{{\displaystyle \underset{\delta_c}{\overset{\infty }{\int }} \exp \left[-\varepsilon \left(\delta \right)/{k}_B T\right]}\  d\delta}{{\displaystyle \underset{0}{\overset{\infty }{\int }} \exp}\left[-\varepsilon \left(\delta \right)/{k}_B T\right]\  d\delta} $$

where *δ*_*c*_ is the value of critical fluctuation:

8 $$ {\delta}_c={u}_c-{u}_f $$

Consequently, the average time before the bond break, *τ*, at a constant value of the applied force *F*_*f*_ is estimated as:

9 $$ \tau =\frac{\tau_0}{P\left(\delta >{\delta}_c\right)} $$

where *τ*_0_ is the average vibration time.

The value of *τ*_0_ can be found by solving the Lagrange equation for the motion of an atom in the potential field *E*(*u*):

10 $$ \delta t=\sqrt{2 m}{\displaystyle \int \frac{du}{\sqrt{U- E(u)}}} $$

where *m* is the mass of an atom; δ*t* is the time; *U* is the total (kinetic and potential) energy of an atom.

Averaging over the ensemble of states gives:

11 $$ {\tau}_0=\frac{{\displaystyle \underset{0}{\overset{\infty }{\int }}\delta t \exp \left(- U/{k}_B T\right)\  dU}}{{\displaystyle \underset{0}{\overset{\infty }{\int }} \exp \left(- U/{k}_B T\right)\  dU}} $$

For the temperature range 300К–2000К the average vibration time is *τ*_0_ = 0.042 ± 0.001*ps* (DFT-findings for *E*(*u*) were used to determine *τ*_0_).

The value of critical displacement *u*_*c*_ (*δ*_*c*_) in dependence (7) is calculated as the solution of equation:

12 $$ {F}_f={F}_{DFT}\left({u}_c\right) $$

relatively to displacement *u*_*c*_ at given value of applied load *F*_*f*_ (Fig. 2);

where *F*_*DFT*_ is DFT-dependence of force on the contact bond length, which is interpolated by quadratic splines.

To derive the dependence *E*(*u*) for *contact* bond, the procedure of integration of the *F*_*DFT*_ -curve was used. Further, this dependence *E*(*u*) was employed to predict the probability of contact bond break within the model proposed.

## Results and discussion

DFT simulation of an atomic and electronic structure of the investigated nanodevice ascertains its metallic conductivity. The results of optimization of atom position in elementary cell are demonstrated in Fig. 1. The lengths of atomic bonds in chain and in graphene are also presented. Alternation of long and short atomic bonds is observed. Short bonds are highlighted in blue colour, while the contact bonds—in a red one. Contact bond has the maximum length of 1.383 Å. According to the evidence obtained, the considered chain is polyyne. Due to the edge effect, interatomic distances on the boundary of graphene sheet differ from the corresponding distance of 1.426 Å in the ideal graphene. The minimum distance is 1.379 Å, and the maximum distance amounts to 1.439 Å.

**Fig. 3:**
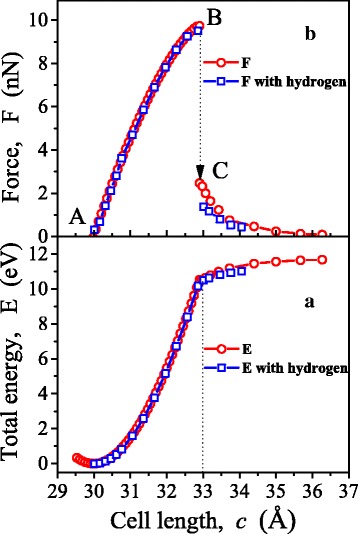
The dependences of total energy, *E*, (*a*) and force, *F*, (*b*) on a cell length *c*, with and without hydrogen

Calculations have shown that the hydrogen termination gave rise to certain decrease in bond lengths in graphene in the vicinity of the terminated atoms (Fig. 1b). The length of the contact bond remained virtually unchanged. It decrease was only 0.004 Å. Respectively, the elastic properties and strength of the *contact* bond also remained practically unchanged (Fig. 3).

**Fig. 4:**
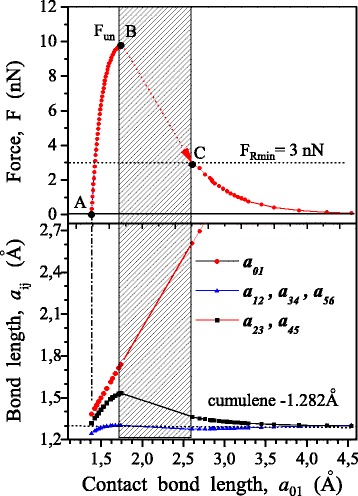
Dependence of force, *F*, acting in a contact bond, on its length, *a*_01_, as well as accompanied changes in the lengths of atomic bonds inside of the carbyne chain

**Fig. 5:**
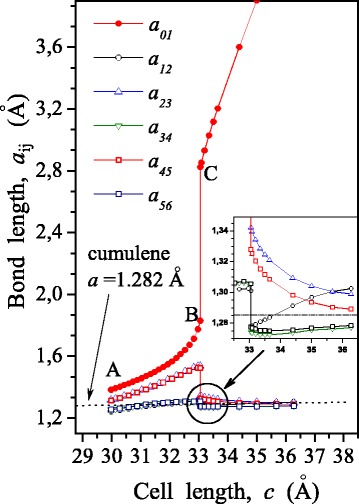
Change in lengths of bonds in carbyne, *a*_*ij*_, at tension of nanoelement: *a*_01_ is the contact bond length

**Fig. 6:**
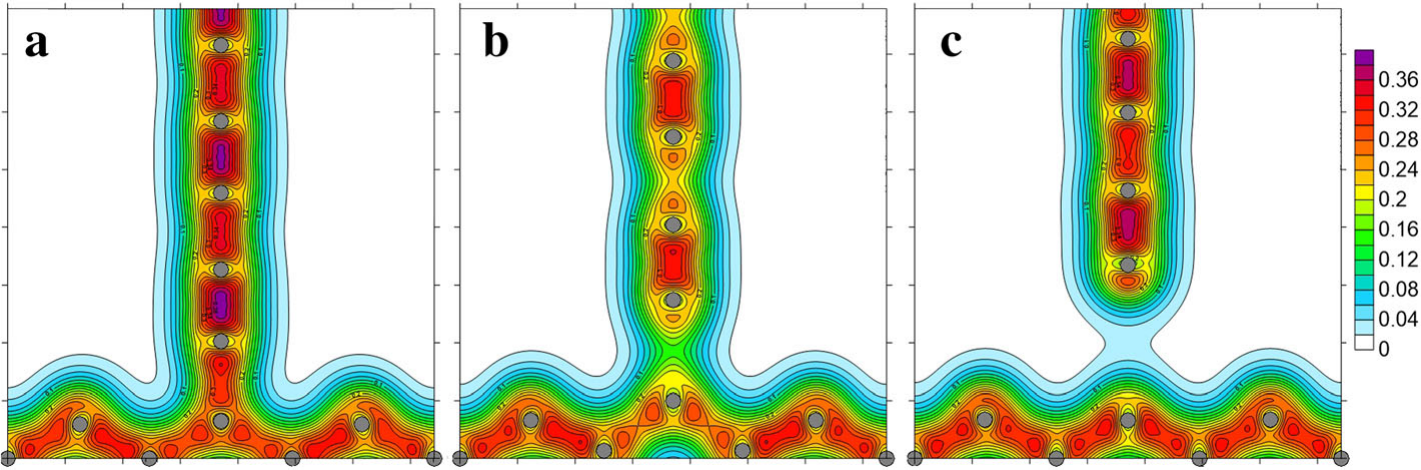
Distribution of the electron density at various stages of tension of nano-elements: ***a*** is the initial state; **b** is the moment of instability; **c** is the moment after restoration of equilibrium (points A, B, C on Fig. 4, respectively)

According to the results DFT calculations, the dependence of total energy *E* on a cell parameter *c* was built (Fig. 3). Here, the dependence of the force *F* on the value of *с* is shown, which was determined by differentiation (the expression (1)). In addition, the dependence of the force acting in the contact bond on its length was constructed (Fig. 4). The regularities of change in bond lengths in chain at tension are shown in Fig. 5. Figure 6 illustrates the distribution of the electron density and atomic structure of the system in the initial state and in key stages of its tension. The key points (“A”, “B”, and “C”) that characterize the behaviour of considered nanodevice during its tension are indicated in these figures.

According to the findings obtained, instability of contact bond occurs in the point “B”. This is evident in both a steep fall in tensile strength of the chain *F* (Fig. 3a) and an increase in the contact bond length *a*_01_ (Fig. 4). This increase in *a*_01_ is accompanied by an abrupt decrease in the interatomic distances in the chain (Fig. 5). Elastic strain energy released at that is spent to perform the work of internal forces for moving the atom of contact bond from a point «B» to a point «C». However, in this case, the magnitude of this energy is not be enough for complete the chain break, so, at point «C» equilibrium between the applied force and the force acting in the contact bond was restored, i.e., at this point the integrity of a nanodevice still maintains. Quantitatively, this is demonstrated in the diagram of dependence of the force on the contact bond length (Fig. 4). Spatial distribution of the electron density in the point “C” is given in Fig. 6c. It is clearly seen that the edge atom of chain still interacts with graphene. Data in Fig. 6b and c illustrates the effect of stress relaxation in the chain after instability of a contact bond (point “C”). It should be emphasized that after a complete contact bond break, a change occurs in the structure of the carbyne chain. The chain forms, which has cumulene structure in it central part. This agrees well with the data on isolated chains obtained earlier [5]. In other words, the transition from polyyne - to cumulene-structure is observed. The latter is quite difficult to be illustrated graphically within a total legend for the electron density, but it follows directly from the data on change in interatomic distances in the carbyne chain given in Figs. 4 and 5.

Thus, the contact bond instability due to thermal fluctuations induces the release of elastic energy accumulated in the loaded crystal, which facilitates the chain break. Herein lays the nature of synergism of the temperature and force effect on stability of the carbyne nanodevice. This phenomenon has a crucial influence on the stability and lifetime of the considered nanodevice under thermo-force loading. Furthermore, starting with a certain level of applied load, synergism can cause a change in kinetics of the contact bond breaking. This should occur when the value of the applied load *F*_*f*_ becomes greater than the value of bonding force of the contact atom with graphene, *F*_*R*_, after relaxation. In this case, instability of the atomic interaction, induced by thermal fluctuations, becomes both a necessary and *sufficient* condition for atomic bond break.

The value of *F*_*R*_ depends on the level of elastic strain energy accumulated in the system. It varies from ${F}_R^{\max }={F}_{un}$ in the absence of mechanical load (*F*_*f*_ = 0) to the minimum value ${F}_R^{\min }=3$ nN, (Fig. 4) at the maximum load *F*_*f*_ = *F*_*un*_. In a first approximation the dependence for *F*_*R*_ is the following (see Appendix):

13 $$ {F}_R\approx \sqrt{F_{un}^2-\alpha {F}_f^2} $$

where for the considered nanodevice *α* = 0.91.

According to (13), the critical value of load ${F}_f^{*}$, beginning with which, the bond instability due to thermal fluctuations results in its break, is defined as:

14 $$ {F}_f^{*}\ge \frac{F_{un}}{\sqrt{1+\alpha}} $$

In our case ${F}_f^{*}\ge 0.72{F}_{un}$.

**Fig. 7:**
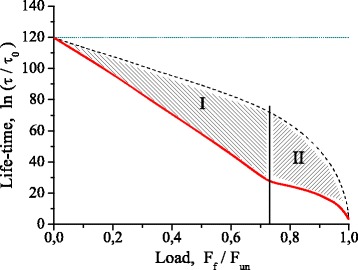
Lifetime dependence on load at T = 600 K: *τ*_0_ = 0.042 ps is the average period of atom vibration; *F*_*f*_ is the value of applied load; *F*_*un*_ is the ultimate tensile strength of a contact bond; I is the region where thermal fluctuations *only initiate* the contact bond break; II is the region where thermal fluctuations are both necessary and sufficient condition for failure; Solid line is the service time with account for the effect of internal forces at release of elastic strain energy. Dash line is the service time without account for above effect. Dot line is the service time by Arrhenius model (calculation by (15))

Figure 7 demonstrates the lifetime dependence on relative applied load at the temperature of 600К.

The dashed curve is obtained without considering of stresses accumulated in the nanodevice, the solid one is obtained taking into account the work performed by these stresses, after the contact bond instability; therefore, steep decrease in the lifetime of nanodevice at transition from the dashed curve to the solid one is a quantitative measure of considered phenomenon of the synergism. Regions «I» and «II» differ in the failure kinetics. In the region «I» the fluctuation-induced instability is necessary but not sufficient condition for the contact bond break. In the region «II» the potential energy accumulated in the nanodevice is sufficient to fluctuation-induced instability of the contact bond (*δ*_*c*_ = *δ*_*un*_, Fig. 2), resulted in its break. At the point of transition from the region “I” into the region “II” the lifetime of nanodevice at *T* = 600 K decreases from 4.6 × 10^10^ years to 0.05 s. This is a clear manifestation of synergism of the effects of temperature and mechanical loading.

**Fig. 8:**
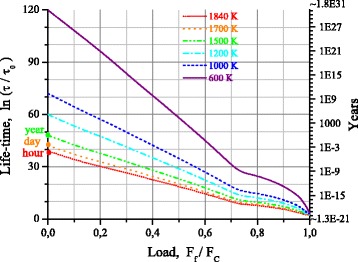
Lifetime dependence on load over the wide temperature range

Figure 8 presents dependences of the waiting time of the contact bond break on the value of relative load for five temperatures. These dependences are obtained within the framework of the suggested model. Data on the diagram of a contact bond strain (Fig. 4) was used for calculation. The values of time before break for the unloaded chain, calculated in the Arrhenius model approximation, are also plotted here:

15 $$ \tau ={\tau}_0 \exp \left({E}_{cog}/ kT\right) $$

where *τ*_0_ = 0.042 ps, and *E*_*cog*_ = 6.44 eV.

As it follows from the calculations, the lifetime of carbyne-based nanodevice is sufficient for application at temperatures not higher than 600 K and loads not exceeding half of tensile strength of the contact bond. At mechanical loads not exceeding 30% of the ultimate one, considered nanodevice can operate also at a temperature of 1000°K. In the unloaded state such nanodevice has a level of thermal stability, sufficient for application up to the temperatures of order 1200–1400 K.

## Conclusions

1. The phenomenon of synergism of both temperature and force effects has a crucial influence on the thermo-mechanical stability of carbyne-based nanodevice in the form of graphene sheets connected by carbyne chain. The reason for this phenomenon consists in the fact that the contact bond instability induced by thermal fluctuations results in release of elastic energy accumulated in the chain, which is spent for the work of internal forces, facilitating the contact bond break.
2. Starting from a certain critical level of this energy, its value becomes sufficient to ensure a contact bond break after its instability, i.e., bond instability due to the thermal fluctuations becomes not only a necessary but also a *sufficient* condition for its break. For the considered nanodevice, this condition starts to be realised when the load value reaches 72% of tensile strength of the contact bond.
3. The suggested fluctuation model of thermo-mechanical stability enables to predict the stability or lifetime of carbyne-based nanodevices, based only on the findings of DFT-calculations, i.e. without employment of empirical parameters.
4. Carbyne-based nanodevices, consisting of two graphene sheets connected by ten-atom carbyne chain, in the unloaded state has a level of thermal stability, sufficient for application up to the temperatures of order 1200–1400 K. At presence of the mechanical loads, not exceeding 30–50% of the contact bond strength, the upper bound of its operational temperature should not exceed the values of 600–1000 K, respectively.

## Appendix

### Calculation of the value *F*_*R*_

The value of *F*_*R*_ is determined by the level of potential energy accumulated in nano-element, so, it depends on the value of applied mechanical load *F*_*f*_. This dependence can be derived from the condition that the energy accumulated in nano-element is spent for moving of the contact bond atom from the point “B” to the point “C” (Fig. 4):

1A $$ {\displaystyle \sum_{i=2}^N\left({\displaystyle \underset{0}{\overset{{u_i}_f}{\int }} Fdu}\right)={\displaystyle \underset{0}{\overset{u_{bc}}{\int }} Fdu}}, $$

where *N* is the number of bonds in nano-element; *u*_*if*_ is the change in length of *i*th bond (*i* = 1 corresponds to the contact bond) at tension; *u*_*bc*_ is change in length of the contact bond after instability.

In general, using the dependence “force vs displacement”, is possible to ascertain a relationship between *F*_*R*_ and the applied force *F*_*f*_.

Use of the first linear approximation for *F* enables to obtain an extremely simple expression for *F*_*R*_:

2A $$ {F}_R\approx \sqrt{F_{un}^2-\alpha {F}_f^2} $$

where *F*_*un*_ is the force of instability of the contact bond (Fig. 4); *α* is the coefficient, which is determined from the data of DFT-calculations (*α* = 0.91):

3A $$ \alpha =\frac{F_{un}^2-{F}_{R \min}^2}{F_{un}^2} $$

where *F*_*R* min_ is the minimum value of force *F*_*R*_ (Fig. 4).

Use of the linear approximation for *F* = *f*(*u*) for all bonds except the contact one (the left part of expression (1A)), is quite reasonable, since till the moment of the contact bond instability, all other bonds are stretched almost linearly. Calculations indicate that the use of this approach for the right side of expression (1A) gives a certain underestimation of *F*_*R*_ for the values of *F*_*f*_ close to zero (*F*_*f*_ ≤ 0.1*F*_*un*_).

The increase in *F*_*R*_ with decreasing in load *F*_*f*_ is accompanied by a corresponding decrease in the distance between the points “B” and “C” along the X axis (Fig. 4). To determine abscissa of the point “C” for a found value of *F*_*R*_ *,* the strain dependence *F*_*DFT*_(*u*) for the contact bond was approximated by cubic splines.
